# The impact of health literacy environment on patient stress: a systematic review

**DOI:** 10.1186/s12889-020-08649-x

**Published:** 2020-05-24

**Authors:** John Yeh, Remo Ostini

**Affiliations:** Faculty of Medicine, The University of Queensland, Herston, Queensland 4006 Australia

**Keywords:** Environment, Health facilities, Health literacy, Patients, Stress

## Abstract

**Background:**

There exists little literature on situational health literacy - that is, how an individual’s health literacy varies across different health literacy environments. However, one can consider the role of stress when examining the relationship between health situations and decision-making ability, and by proxy health literacy. The aim of this study was to assess the strength of the evidence on the relationship between health situations and patient stress, considered in the context of health professional perception, and determine what health situations act to influence patient stress.

**Methods:**

A systematic review of English articles using PubMed, PsycINFO, CINAHL and Embase databases was conducted. Search terms focused on ‘patient’, ‘stress’, and ‘health care situations’. Only peer-reviewed original research with data on patient stress in the context of a health facility environment was included. Studies were screened and critically appraised by both authors. Study elements for extraction were defined by RO and extracted by JY.

**Results:**

Twenty-four studies were included for narrative synthesis. Patients in Intensive Care Units were more stressed about factors relating to their physical discomfort, with some agreement from health care professionals. Parents of children in Intensive Care Units were more concerned with stressors relating to their child’s appearance and behaviour, and alteration in their parental role. Few studies examined health settings other than Intensive Care Units, and those that did varied greatly in terms of study design and population characteristics, lacking generalisability.

**Conclusions:**

Overall, the findings of what patients find most stressful in Intensive Care Units can guide health care professionals practicing best practice care. However, the evidence on how patient stress is influenced by non-Intensive Care Unit health care settings is weak. Further research is needed to enhance current understanding of the interaction between patient stress and health care environments in both hospital and primary care settings.

## Background

### Health literacy

Health literacy can be defined as “the degree to which individuals have the capacity to obtain, process, and understand basic health information and services needed to make appropriate health decisions” [1]. Its importance is well established. Not only is low health literacy associated with poor health for individuals and poor financial outcomes for health systems [2, 3], it is also highly prevalent both in Australia and internationally [1, 4].

Increasingly, health literacy is recognised as dynamic, varying depending on the context and characteristics of individuals, environmental and social factors, and the demands and burdens placed on individuals [1, 5–12]. Indeed, a critical component of health literacy is the health literacy environment, which is the “infrastructure, policies, processes, materials, people and relationships that make up the health system and have an impact on the way that people access, understand, appraise and apply health-related information and services” [13]. However, despite a few key studies [14, 15], there is still little current literature on situational health literacy – that is, how an individual’s health literacy varies across different health literacy environments.

Stress can be considered when examining the relationship between health situations and decision-making ability, and by proxy health literacy. Much of the literature suggests that stress impairs decision-making ability [16, 17], through several mechanisms. Firstly, high stress levels have been found to cause inadequate consideration of alternatives, resulting in dysfunctional strategy use [16]. Additionally, there is an over-reliance on intuitive decision-making rather than strategic choice, reflecting an insufficient adjustment from automated responses [16]. Furthermore, stress triggers altered feedback processing, causing stressed individuals to make more disadvantageous choices [16]. Given the clear influence of stress on decision-making ability [16, 17], the focus of the current study is on what health situations act to influence patient stress.

### Health situations and stress

Environmental factors are known to play a large role in increasing or decreasing patient stress [18]. Much of the literature focuses on stressors, both physical and psychological, associated with hospitalisation. These include lack of natural light, increased noise levels, presence of unwelcome smells or other sensations, absence of clocks, and a perception of crowding by unfamiliar people [19–21]. Intensive Care Units (ICUs) especially are known to be stressful environments [19], with specific stressors including experiencing pain, disruption of sleep-wake patterns, and intubation [22, 23]. A perceived lack of control over these physical environmental factors also increases patient stress [21, 24]. In addition, hospital patients are stressed by their inability to obtain desired information, and their fear of an unknown or serious illness [24]. Furthermore, decreased social support, such as with loss of contact with family and friends, and financial worries associated with hospitalisation and illness, have been identified as stressful for patients [19, 22, 24].

Stress-reducing environmental factors have also been identified. Viewing plants and nature has been shown to decrease patient stress [25], an effect which was reproduced by viewing photographs of plants or nature in a hospital waiting room [26]. In addition, environmental interventions that counter stress-inducing factors are thought to reduce patient stress [18]. Recommendations have been made for single-bed rather than multi-bed rooms, and elimination of noise sources, as these would lead to improved sleep [18]. Similarly, administrative and procedural information, external building cues, local information systems, and global structural redesign, have also been recommended as these interventions reduce spatial disorientation, and consequently decrease patient stress [18, 27]. These recommendations appear to also apply beyond hospitals to the primary care setting [28].

There are also other aspects of health situations that influence patient stress. These include the provider-consumer interaction – that is, what physicians say and how they deliver this information [29]. In particular, taking time, empathising with patients, and overall effective communication skills have been shown to help reduce patient stress [29, 30]. Additionally, the broader social, economic, and psychosocial contexts of stress can be considered. Constant exposure to socially and economically challenging environments has been shown to increase stress [31], while strong social support networks protect against stress and other environmental threats to health [31, 32].

Clearly, many health situations affect patient stress, potentially influencing decision-making ability and by proxy health literacy. However, the cumulative strength of the evidence on this relationship between health situations and patient stress has not been evaluated. The aim of this systematic review is to assess the strength of this evidence, and determine what health situations act to influence patient stress. In doing so, this study will also inform our understanding of health situations in supporting or constraining health literacy.

## Methods

The study design of this systematic review was guided by the PRISMA Statement [33] (Appendix 1). Studies were identified by searching through the electronic databases PubMed, PsycINFO, CINAHL and Embase, with coverage from 1997 to February 2017. This timeframe was selected to provide a long enough period to comprehensively review the research, whilst maintaining the relevance of the health care context to contemporary practice. No grey literature was reviewed, and the reference lists of included articles were not searched for additional studies.

The search terms comprised of three components: patient; stress; and health care environment. Index terms were used when possible as appropriate for each database, and synonyms were included. Terms relating to stress of health care professionals or staff, and mental disorders of patients, were excluded from the search. In addition, the search was limited to journal articles published in English since 1997. The full search strategy is provided in Appendix 2.

**Fig. 1:**
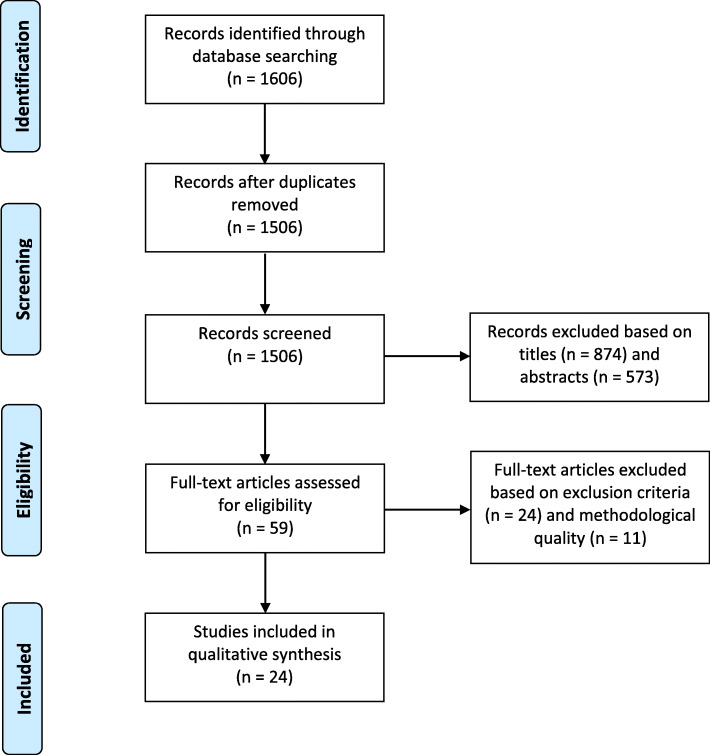
Flow diagram of study selection process, including formal search, screening, application of inclusion and exclusion criteria, and critical analysis, with the number of articles included and excluded at each step. Adapted from the PRISMA Statement [33]

The study selection process is summarised in Fig. 1. Articles identified through the search process were screened by JY based on titles alone, removing articles that were not relevant to the current study question. The abstracts of the remaining articles were then assessed independently by JY and RO, applying the pre-determined inclusion and exclusion criteria (Table 1). Articles without abstracts were removed at this point. Disagreements regarding article inclusion or exclusion were resolved by a third reviewer, NA. Articles that were evaluated as sufficiently rigorous were included for critical appraisal. This process was completed independently by JY and RO, using an appraisal template adapted from Bush et al. [34] (Additional file 1). Articles of lower methodological strength, generally corresponding to a score of 0 or failing to score 2 in any of Appraisal Questions 1–5, were excluded. Discrepancies regarding the eligibility of articles at this stage were resolved by discussion between the two reviewers until agreement was reached. Eligible articles were included in the qualitative analysis.

**Table 1:** Study inclusion and exclusion criteria

Inclusion	Exclusion
Peer-reviewed original research (including systematic reviews)	Not original research (eg. Overview, descriptive review, editorial, opinion piece, conference abstract/paper, thesis, books/book chapters)
English	Not English
Explicit measure of patient stress (or parental stress if the patient is a child) in the context of a health facility environment in which stress was measured	No measure of patient stress Not in context of health facility environment Stress as a result of a diagnosis Stress as a result of individual doctor-patient interactions Stress as a result of a procedure (eg. Mechanical ventilation, MRI) Stress of family (other than parents) of patients Interventions that target only stress without examining the environment

Data extraction items were defined by RO and extracted by JY from eligible articles. These data items included: study source, design, country, population characteristics, health care setting, measured variables, intervention, and outcomes. Due to the nature of the types of studies included, a narrative synthesis, rather than meta-analysis, was the more appropriate method of analysis.

## Results

A total of 1506 unique articles were identified through the database searches conducted. Of these, 874 articles were excluded based on title-scanning as they were not addressing the research question. A further 573 were excluded based on abstract-scanning as they did not meet the inclusion and exclusion criteria. Full texts of the remaining 59 articles were critically appraised, and 24 were further excluded based on exclusion criteria whilst 11 were excluded after formal appraisal of methodological strength. The included articles received appraisal scores ranging from six to nine, out of a possible twelve. Overall, 24 articles were assessed to be eligible for data extraction and narrative synthesis, and were included in this systematic review (Fig. 1).

The study designs of included research were mostly descriptive – of the 24 studies, nineteen (79%) were cross-sectional surveys, one used a descriptive qualitative design, and only four were experimental. Included studies were also internationally diverse. Nine studies were set in Asia, eight in Europe, six in the USA, two in Brazil, and one in New Zealand. Furthermore, all studies were conducted in hospital settings, with the majority (71%) set in ICUs (nine adult, eight paediatric/neonatal). Regarding participants, eight studies focused on parents and sixteen on patients, with five of the latter also including health care professionals. Most participants were adults, with only Causey et al. [35] recruiting children and adolescents, and Yeh et al. [36] recruiting patients as young as fifteen years. Finally, the measured variable, stress, was assessed with validated tools in most studies (92%), while Larsen, Larsen and Birkelund [37] conducted participant observation and interviews, and Lilja, Ryden and Fridlund [38] measured serum cortisol (Table 2).

**Table 2:** Summary of study characteristics, including design, country, population and setting, and measured variables

Source	Design	Country	Population & Setting	Measured variables
*Patient stressor scales*				
Biancofiore et al. 2005 [22]	Cross-sectional survey	Italy	104 orthotopic liver transplant patients, 103 elective major abdominal surgery patients, 35 ICU nurses & 21 ICU physicians, in a 10-bed post-surgical ICU	ICU Environmental Stressor Scale
Causey et al. 1998 [35]	Cross-sectional survey	USA	40 child & adolescent patients admitted to Ackerly Psychiatric Inpatient Unit, at major medical hospital	Child and Adolescent Psychiatric Hospitalisation Stressor Survey
Dias, Resende & Diniz 2015 [19]	Cross-sectional survey	Brazil	60 patients in 2 hospital ICUs (30 each)	Assessment Scale for Stressors in the Intensive Care Unit (Brazilian-Portuguese version of Environmental Stressor Questionnaire)
Hweidi 2007 [39]	Cross-sectional survey	Jordan	165 patients in 3 CCUs	ICU Environmental Stressor Scale (Arabic version, 42 items)
Lam Soh et al. 2008 [40]	Cross-sectional survey	Malaysia	70 ventilated adult patients in 4 ICUs (general ICU, urology ICU, CCU)	Modified Environmental Stressor Questionnaire (translated to Bahasa Malaysia)
Novaes et al. 1999 [23]	Cross-sectional survey	Brazil	50 sets of adult patients, respective relatives & health team professionals in a general adult ICU	ICU Environmental Stressor Scale (translated to Portuguese, 40 items)
Pang & Suen 2008 [41]	Cross-sectional survey	China	60 patients & 54 critical care nurses in a hospital ICU	ICU Stressor Questionnaire (Chinese) (translated from Environmental Stress Questionnaire)
Samuelson, Lundberg & Fridlund 2007 [42]	Cross-sectional survey	Sweden	313 adult patients who had been intubated and mechanically ventilated in 2 general ICUs	ICU Stressful Experiences Questionnaire
So & Chan 2004 [43]	Cross-sectional survey	China	50 patients & 92 nurses directly involved in the care of patients in 3 CCUs	ICU Environmental Stressor Scale (Chinese version, 42 items)
Yava et al. 2011 [44]	Cross-sectional survey	Turkey	155 adult patients & 152 ICU nurses in ICUs of 2 hospitals	ICU Environmental Stressor Scale (translated to Turkish)
Yeh et al. 2009 [36]	Cross-sectional survey	Taiwan	2642 patients, 15 years or older, with end-stage renal disease on dialysis for at least 3 months, at 5 medical centres, 5 regional hospitals, 10 community hospitals & 7 independent haemodialysis centres	Haemodialysis Stressor Scale (Chinese adaption)
*Parental stressor scales*				
Board & Ryan-Wenger 2003 [45]	Cross-sectional survey	USA	31 mothers with child in PICU & 32 mothers with child in GCU, in large 311-bed children’s hospital in Midwest	PSS: PICU
Board 2004 [46]	Cross-sectional survey	USA	15 fathers with child in PICU & 10 fathers with child in GCU, in large children’s hospital in Midwest	PSS: PICU
Franck et al. 2005 [47]	Cross-sectional survey	UK & USA	257 parents of infants admitted to NICU (184 mothers, 73 fathers), in 9 UK NICUs & 2 US NICUs	PSS: NICU
Ichijima, Kirk & Hornblow 2011 [48]	Cross-sectional survey	New Zealand & Japan	121 parents of children requiring NICU hospitalisation, in Christchurch NICU (*n* = 61) & Tokyo NICU (*n* = 60)	PSS: NICU (modified version, ‘communication with staff’ excluded, translated to Japanese)
Lee et al. 2005 [49]	Cross-sectional survey	USA	55 Chinese or Chinese-American parents of 31 infants in ICU, in tertiary NICU, PICU & cardiac ICU of 3 teaching hospitals	PSS: Infant Hospitalisation-modified (translated to Chinese); Structured interview
Miles et al. 2002 [50]	Cross-sectional survey	USA	69 mothers (31 Black, 38 White) of infants with serious life-threatening illness, in NICU, PICU & selected wards of tertiary care hospital in Southeast	PSS: Infant Hospitalisation (adapted from PSS: NICU)
Nizam & Norzila 2001 [51]	Cross-sectional survey	Malaysia	94 parents or primary caregivers with children admitted to PICU or PHDU	PSS: PICU (translated to Malay)
Reid & Bramwell 2003 [52]	Cross-sectional survey	UK	40 mothers with preterm infants in NICU	PSS: NICU
*Qualitative and experimental studies*				
Larsen, Larsen & Birkelund 2014 [37]	Descriptive	Denmark	20 adult Danish-speaking hospitalised cancer patients, in large university hospital & smaller regional hospital	Participant observation; Individual semi-structured interviews
Beukeboom, Langeveld & Tanja-Dijkstra 2012 [26]	Controlled trial	The Netherlands	457 patients (160 ‘no plants’, 150 ‘real plants’, 147 ‘posters’), in Radiology Department waiting room	DV: Experienced stress level measured by combined score on Profile of Mood states (shortened version) & State Trait Anxiety Inventory (Dutch, abridged); Perceived attractiveness of room Intervention: Exposure to nature (real plants vs. posters vs. no plants)
Cantekin & Tan 2013 [53]	Controlled before and after	Turkey	100 patients receiving haemodialysis treatment (50 control, 50 experimental), at haemodialysis units of 2 hospitals	DV: Perceived stressors measured by Hemodialysis Stressor Scale Intervention: Music therapy (Turkish art music songs)
Lilja, Ryden & Fridlund 1998 [38]	Pre-post study	Sweden	44 breast cancer patients (22 intervention, 22 control) & 50 total hip replacement patients (22 intervention, 28 control), in 400-bed hospital in south-west Sweden	DV: Stress conceptualised by serum cortisol measured 1 day pre-op, day of surgery, day 1 post-op & day 3 post-op Intervention: Preoperative information from anaesthetic nurse
Muller-Nordhorn et al. 2006 [54]	Pre-post study (parallel)	Germany	138 adult patients (64 inpatient, 74 outpatient), with indication for elective pacemaker implantation or system change, in teaching hospital or outpatient clinic	DV: Subjective stress measured by German Short Questionnaire on Current Stress Intervention: Pacemaker implantation

*ICU* intensive care unit; *CCU* critical care unit; *PICU* paediatric intensive care unit; *NICU* neonatal intensive care unit; *GCU* general care unit; *PHDU* paediatric high dependency unit; *PSS* parental stressor scale; *DV* dependent variable

### Patient and parental stressors in ICU

Considering the nine studies that ranked patient stressors in ICU [19, 22, 23, 39–44], the items from the ICU Stressor (or similar) scales that were rated highly most frequently related to physical discomfort. As examples, top ten stressors included ‘being in pain’ in all studies, and ‘being unable to sleep’ in all but one study. Overall, seven of thirteen (54%) items in the physical discomfort subscale were listed as top ten in two or more studies. In contrast, only 35% of stressors relating to psychological distress, and 36% stressors relating to the ICU environment, were ranked highly in at least two studies. No stressors relating to treatment procedures were listed more than once in the top ten (Table 3).

**Table 3:** Summary of main study outcomes

Source	Scores (RO, JY)	Main Study Outcomes
*Patient stressor scales*		
Biancofiore et al. 2005 [22]	6, 8	ICU-related stressors evaluated differently by study groups (p < 0.001) Top 10 stressors for OLT patients (elective abdominal surgery patients, nurses, physicians): 1) being unable to sleep (2, 6, 3), 2) being in pain (3, 2, 1), 3) having tubes in nose/mouth (3, 2, 1), 4) missing husband/wife (5, 9, 9), 5) seeing family & friends only a few minutes a day (1, 7, 11), 6) being tied down by tubes (7, 8, 6), 7) being thirsty (6, 11, 4), 8) hearing the heart alarm (11, 12, 8), 9) having no control over oneself (8, 5, 5), 10) uncomfortable bed/pillow (10, 15, 16) Orthotopic liver transplant (52%) & major abdominal surgery patients (61.4%) used score of 1 (not stressful) more frequently than nurses (19.1%) & physicians (23.4%) (*p* < 0.001); nurses & physicians more frequently used scores of 2, 3 & 4
Causey et al. 1998 [35]	8, 7	Highest rated items: 1) being away from and missing all your friends, 2) being away from and missing your family, 3) not being able to exercise, play, or go outside for fresh air, 4) not having enough time to visit or talk with your family and friends, 5) not knowing how long you will be in the hospital, 6) being in a place where all the doors are locked, 7) not being able to have your own things from home, 8) not being able to do the things you normally do at home, 9) being watched too much by staff, 10) not feeling you know enough from your doctor about things that concern you Subscale rankings: 1) family/friends separation, 2) loss of autonomy, 3) psychiatric setting, 4) therapeutic/staff interactions, 5) rules and authority, 6) stigmatisation
Dias, Resende & Diniz 2015 [19]	8, 9	Coronary ICU Major stressors: 1) being in pain, 2) being unable to fulfil family roles, 3) being bored, 4) not being able to sleep, 5) having financial worries, 6) not being in control of yourself, 7) not being able to communicate, 8) hearing people talk about you, 9) being afraid of catching AIDS, 10) only seeing family and friends for a few minutes each day Postoperative ICU Major stressors: 1) being in pain, 2) being unable to fulfil family roles, 3) not being able to communicate, 4) not being able to sleep, 5) being afraid of catching AIDS, 6) having no privacy, 7) being bored, 8) being in a room that is too hot or too cold, 9) having lights on constantly, 10) not being able to move your hands or arms because of IV lines
Hweidi 2007 [39]	7, 8	Top 10 stressors: 1) having tubes in your nose or mouth, 2) being in pain, 3) not able to sleep, 4) hearing the buzzers and alarms from the machinery, 5) being thirsty, 6) not being in control of yourself, 7) unfamiliar and unusual noises, 8) being tied down by tubes, 9) watching treatment being given to other patients, 10) being awakened by nurses
Lam Soh et al. 2008 [40]	6, 8	Top 10 stressors: 1) in pain, 2) stuck with needles, 3) bored, 4) missing husband/wife, 5) room too hot/cold, 6) cannot sleep, 7) cannot move hands/arms because of IV line, 8) tubes in your nose/mouth, 9) staring at tiles in the ceiling, 10) thirsty
Novaes et al. 1999 [23]	8, 8	Top stressors for patients (relatives, team): 1) being in pain (1, 1), 2) being unable to sleep (4, 4), 3) having tubes in nose and/or mouth (2, 2), 4) having no control on oneself (6, 19), 5) being tied down by tubes (3, 3), 6) receiving no explanations about the treatment (11, 9), 7) being unable to move the hands or arms because of IV tubes (5, 21), 8) not knowing when things will be done to you (14, 16), 9) being stuck with needles(19, 7), 10) being thirsty (12, 18) Significant difference between scores rated by patients & health care professionals (*p* = 0.018) No difference between patients & relatives (*p* = 0.185), or relatives & health care professionals (*p* = 0.114)
Pang & Suen 2008 [41]	7, 8	Top stressors for patients (nurses): 1) fear of death (1), 2) being pressurised to consent to treatment (4), 3) being in pain (6), 4) not knowing the length of stay in ICU (18), 5) not being able to communicate (3), 6) fear of other hospital-transmitted diseases (25), 7) not having treatments explained to you (12), 8) financial worries (11), 9) having tubes in your nose or mouth (5), 10) unfamiliar and unusual noises (16)
Samuelson, Lundberg & Fridlund 2007 [42]	9, 8	Top 10 ICU stressors: 1) trouble sleeping, 2) being thirsty, 3) being restricted by tubes and lines, 4) being in pain, 5) trouble falling asleep, 6) difficulty swallowing, 7) spells of terror or panic, 8) not being able to sleep, 9) not being in control, 10) feeling fearful
So & Chan 2004 [43]	8, 8	Top 10 stressors for patients (nurses): 1) being tied down by tubes (1), 2) not being in control of yourself (9), 3) not being able to sleep (11), 4) hearing the buzzers and alarms from the machinery (4), 5) being thirsty (40), 6) being in pain (6), 7) not knowing when to expect things will be done to you (8), 8) having your BP taken often (26), 9) missing your husband or wife (19), 10) having nurses be in too much of a hurry (18)
Yava et al. 2011 [44]	8, 8	Top 10 stressors for patients (nurses): 1) fear of death (1), 2) being thirsty (13), 3) being in pain (2), 4) not being able to sleep (4), 5) having tubes in your nose or mouth (3), 6) hearing other patients cry out (8), 7) being restricted by tubes/lines (11), 8) not being able to move your hands or arms because of IV lines (12), 9) uncomfortable bed or pillow (23), 10) having lights on constantly (18)
Yeh et al. 2009 [36]	8, 9	Patients across 3 types of facility (Veterans/Army (VA); For Profit (FP); Religious Affiliated (RA)) were statistically significantly different in what they perceived as stressful: RA higher stress in physical symptoms (F = 15.01, p < 0.001), dependency on medical staff (F = 19.72, *p* < 0.001), role ambiguity (F = 6.80, *p* = 0.001), blood vessel problems (F = 27.70, p < 0.001) VA higher stress in food & fluid restriction (F = 4.49, p = 0.01; mean = 5.27), dependency on medical staff (F = 19.72, p < 0.001) than FP
*Parental stressor scales*		
Board & Ryan-Wenger 2003 [45]	7, 7	Most frequently experienced maternal stressors (> 90%): PICU: (100%) total experience is stressful, injections/shots, sudden sounds of monitor alarms, seeing heart rate on monitor, sound of monitors and equipment; (97%) putting needles in child; (90%) too many different people talking to me, tubes in my child GCU: (97%) putting needles in child; (95%) acting or looking as if in pain; (90%) crying or whining
Board 2004 [46]	8, 6	Mean PSS: PICU (2.06 (SD 0.78)); GCU (1.47 (SD 0.86)) no significant difference Most frequently experienced paternal stressors (> 90%): PICU: (100%) tubes in my child; (93%) putting needles in my child for fluids/procedures or tests, not knowing how best to help my child during this crisis GCU: (90%) putting needles in my child for fluids, procedures or tests
Franck et al. 2005 [47]	8, 8	Metric 1 (stress occurrence) subscale ranking UK (US): 1) parent role alteration (1), 2) infant behaviour and appearance (2), 3) sights and sounds (4), 4) staff behaviour and communication (3) Metric 2 (overall stress) subscale ranking UK (US): 1) parent role alteration (1), 2) infant behaviour and appearance (2), 3) sights and sounds (3), 4) staff behaviour and communication (4)
Ichijima, Kirk & Hornblow 2011 [48]	7, 8	Christchurch maternal stress related to sights & sounds associated with feeding status of infants (p = 0.01): stress higher when tube feeding only Tokyo maternal stress related to sights & sounds negatively correlated with total hours they visited unit (*p* = 0.004) & infant birth weight (*p* = 0.025)
Lee et al. 2005 [49]	9, 8	Subscale rankings: 1) child appearance, 2) parental role, 3) HCP’s communication, 4) HCP’s behaviour, 5) ICU environment Structured interviews - 7 themes: Lack of confidence; Self-blame; Worry about upsetting own parents; Lack of resources; Stress related to communication issues; Stress related to cultural issues; Other issues: changing bed spaces/hospital units, difficulty accessing doctors
Miles et al. 2002 [50]	8, 8	Subscale rankings: 1) infant appearance and behaviour, 2) parental role alteration, 3) sights and sounds Top 5 stressors: Black mothers: 1) breathing problems, 2) seeing child in pain, 3) can’t protect from pain, 4) can’t respond to me, 5) separated from baby White mothers: 1) seeing child in pain, 2) breathing problems, 3) can’t protect from pain, 4) separated from baby, 5) can’t respond to me
Nizam & Norzila 2001 [51]	7, 8	Subscale rankings: 1) parental roles, 2) child’s behaviour and emotional response, 3) sight and sound, 4) child’s appearance, 5) procedure, 6) staff’s communication, 7) staff’s behaviour No significant difference of means between parents of 2 units Fathers higher than mothers in staff’s communications (3.15 vs 2.50, *p* < 0.017) Staff’s communication higher if child not ventilated prior (2.94 vs 3.26, *p* = 0.05)
Reid & Bramwell 2003 [52]	7, 8	Subscale ranking: 1) relationship with infant, 2) appearance and behaviour, 3) sights and sounds, 4) staff behaviours and communication (many items n/a in > 2/3 participants – excluded from further analyses) Younger mothers, less education, poorer SES - more stress on environment subscale, but not significant on multiple regression ‘Sights & sounds’ had moderate correlation with infant variables: days to full feeds, length of stay
*Qualitative and experimental studies*		
Larsen, Larsen & Birkelund 2014 [37]	8, 9	Themes: Healing & non-healing accommodation; Withholding information due to enforced public privacy; Seeking refuge from fellow patients; Single-bed room or multiple-bed room; Acceptance of & resignation to the hospital environment
Beukeboom, Langeveld & Tanja-Dijkstra 2012 [26]	8, 8	Marginal effect on exposure to nature, F (2,451) = 2.33, *p* = 0.099, np^2 = 0.01; Tukey post hoc test: real plants & posters both lower stress (p’s = 0.04) Real plants vs. posters no difference Mean (SD) experienced stress: no plants = 2.51 (0.87); real plants = 2.27 (0.79); posters = 2.27 (0.86) Partial mediation by perceived attractiveness of room
Cantekin & Tan 2013 [53]	7, 8	Both psychosocial (mean difference 7.4, p < 0.01) and physiological (mean difference 3.7, *p* < 0.001) stress rated lower after music therapy; no mean change in control group Overall stress lower in experimental group (mean difference 12.5, p < 0.01) and higher in control group (mean difference 2.6, *p* < 0.001)
Lilja, Ryden & Fridlund 1998 [38]	7, 7	No significant differences in cortisol seen between intervention & control groups, for both breast cancer & total hip replacement patients
Muller-Nordhorn et al. 2006 [54]	8, 6	In both inpatients & outpatients, subjective stress decreased from pre-op, to day 1, to day 3/4 - no significant differences in stress between groups at any time

*ICU* intensive care unit; *PICU* paediatric intensive care unit; *GCU* general care unit; *HCP* health care professional; *SD* standard deviation

Comparing stressors as ranked by patients with health care professional rankings, shows that some stressors were also ranked consistently highly by health care professionals across studies, such as ‘being in pain’, ‘being tied down by tubes’, and ‘having tubes in your nose/mouth’. However, for the most part, stressors were evaluated differently by patients and health care professionals, with significant differences between scores on stressor scales in some studies [22, 23]. In particular, Biancofiore et al. [22] found that patients used a score of 1 (not stressful) more frequently than health care professionals when rating stressors, while health care professionals tended to use scores of 2, 3 and 4 (increasing stressfulness) more frequently (Table 3).

For parents, stressors relating to the appearance and behaviour of their child, or alteration of their parental role, were ranked most highly in almost all studies. Parental role alteration, and child appearance and behaviour were ranked first and second respectively by parents in studies of Franck et al. [47], Nizam and Norzila [51], and Reid and Bramwell. On the other hand, stressors relating to staff communication and behaviour, and the ICU environment, were consistently at the bottom of parent rankings (Table 3).

### Non-ICU settings

Seven of the 24 studies examined health care environments other than ICU. These included a psychiatric inpatient unit; medical, oncology and surgical wards; haemodialysis units across various medical centres, regional and community hospitals; and a radiology department waiting room. The designs of these seven studies varied greatly, ranging from cross-sectional survey, to qualitative and experimental, the interventions of which showed inconsistent effects (Table 2).

Children and adolescents admitted to an inpatient psychiatric unit appeared to be more stressed by separation from their friends and family, and their loss of autonomy, than by other aspects of the health care environment such as the psychiatric setting and staff interaction [35]. More broadly, stressors were found to be perceived differently by patients depending on the type of health facility. Yeh et al. [36] showed that issues such as physical symptoms, dependency on medical staff, and role ambiguity, were rated higher by patients in ‘Religious Affiliated’ hospitals, while patients in ‘Veterans/Army’ hospitals experienced significantly higher stress in food and fluid restriction, and dependency on medical staff, than patients in ‘For Profit’ hospitals.

Larsen, Larsen and Birkelund [37] found, through interviews with cancer patients, several recurring themes relating to the health care environment. These included the health effects, both positive and negative, of a health care setting, and the implications of (lack of) patient privacy, such as withholding information from doctors, avoiding other patients, and preferring single-bed rooms. Despite these apparent shortcomings of their accommodation, the patients maintained a sense of acceptance of and resignation to the hospital environment.

Several studies investigated whether altering aspects of the health care setting would impact patient stress. Beukeboom, Langeveld and Tanja-Dijkstra [26] tested the effect of exposure to nature on stress, and found that both real plants and posters of plants significantly lowered the stress of patients in a waiting room. This effect was at least partially mediated by the perceived attractiveness of the room. Additionally, music therapy appeared to decrease both psychosocial and physiological stress for patients in haemodialysis units [53]. On the other hand, preoperative information provided by an anaesthetic nurse had no significant impact on patient stress [38]. Only one study compared inpatient and outpatient settings, which found no significant differences in stress between the two groups, in patients awaiting pacemaker implantation [54].

## Discussion

### Summary of evidence

There are several main findings regarding what was identified as stressful about health care settings. Firstly, patients in ICU were consistently more stressed about factors relating to their physical discomfort, than those relating to psychological distress or the ICU environment. Meanwhile, apart from some similarity with stressors relating to physical discomfort, health care professionals placed importance on different stressors to patients, and generally rated them as more highly stressful to patients than did patients. Additionally, parents of children in ICU consistently placed the most importance on stressors relating to their child’s appearance and behaviour, and alterations in their parental role, while being less concerned about stressors relating to staff communication and behaviour, and the sights and sounds of the ICU environment. Overall, the strength of the evidence for these findings is acceptable, and while difficult to apply to non-ICU settings, can be expected to be consistent between ICUs especially given the international diversity of the included studies.

Adult patients in an oncology ward often found their lack of privacy, related to the architecture in multiple-bed rooms, stressful and non-healing [37]. That this finding is not entirely consistent with the above studies, where stressors relating to the health care environment were less worrisome, may be explained by the different study designs and health settings. In addition, children and adolescents in a psychiatric unit appeared to find separation from family and friends, and loss of autonomy, more stressful than staff interactions and the psychiatric environment. However, the evidence for this finding is less robust given no studies other than that of Causey et al. [35] investigated this population and health care setting.

The nature of the health care setting appeared to have variable effect on patient stress. Broadly, the type of health care facility, specifically its ownership, influenced how stressors were perceived by its patients [36]. In contrast, no differences in levels of patient stress were found between inpatient and outpatient health settings [54]. Admittedly, these results are difficult to compare as the studies were very different, in terms of study design, population characteristics, and assessment tool.

The experimental studies found that exposure to nature and plants, and music therapy, decreased patient stress, while pre-operative provision of information had no such reduction on patient stress. These findings, while promising, carry less weight than the previous outcomes. One of the main reasons for this is that across the few included experimental studies, the population, setting, interventions, and measured outcomes all differ. Furthermore, they lack generalizability – for example, the music intervention used by Cantekin and Tan [53] was Turkish art music songs, the effect of which may be difficult to reproduce in other populations. Therefore, the evidence of these outcomes is relatively weak.

This systematic review set out to firstly assess the strength of evidence on the relationship between health care situations and patient stress, and secondly determine what health care situations act to influence patient stress. It has partially met these aims. Certainly, this study confirms that ICUs are indeed stressful environments and produces some detail on specific stressors; however, the evidence for other outcomes in different health care settings is insufficiently robust.

The importance of stress in the relationship between health situations and decision-making ability relates to the way high stress levels result in dysfunctional strategies, over-reliance on intuitive decision-making, and more disadvantageous choices [16]. This implies that patients in stressful health situations such as ICU would have impaired decision-making abilities and likely constrained health literacy. How specific features of these health situations lead to patient stress, and how stressors in these contexts affect decision-making ability are important areas to examine further.

### Limitations

The study has several limitations. Firstly, the review process may have been limited by the search strategy. The diversity of the literature around stress and healthcare made it difficult to develop a strategy with sufficient sensitivity and specificity. This meant that a large variety of articles, often tangential to the topic, were identified, and additionally the risk of missing key articles was increased. This may have been exacerbated by the restriction to only English-language publications. Furthermore, no additional searches were conducted for grey literature, and articles were not drawn from reference lists of included studies, or those citing included studies. This may have introduced a degree of publication bias. Another limitation was the lack of weighted scoring in the appraisal tool, meaning no explicit cut-off value could be set as an inclusion or exclusion criterion.

In addition, a limitation of the data analysis was the diverse nature of the included studies, with study designs, patient populations, health settings, interventions, and outcome measure often differing across studies. Moreover, a narrative synthesis, which was necessary given the types of include studies, cannot provide the same degree of strength of evidence as a meta-analysis. That the main findings of the analysis were drawn mostly from studies set in the ICU also limits the generalizability of the review. One issue specific to these studies is that using the stressor scales as assessment tools presupposes the factors that patients find stressful, and may not account for other relevant stressors.

### Implications

An important next step would be to investigate patient stress in the context of health care settings other than the ICU. These should include not just other aspects of the hospital setting, such as different wards or waiting rooms, but also extend to the primary care setting. While an enhanced primary care environment has been associated with decreased patient anxiety and improved patient satisfaction [28], further research could enhance current understanding of the interaction between patient stress and this environment. As individual stressors are increasingly identified, future research could also investigate the effects of interventions that target these stressors. Ultimately, given the original focus on decision-making and health literacy, future research must directly investigate health literacy levels of patients and how this is influenced by different health care settings. Furthermore, this research should examine other aspects of the health literacy environment, such as relationships, infrastructure, and policies. Studies of this kind will more likely have lasting impact on clinical practice and health care design.

## Conclusions

Overall, this systematic review has revealed that the evidence on how patient stress is influenced by non-ICU health care settings is weak. That being said, this review does show what patients find most stressful in the ICU, and these findings can guide health care professionals practicing best practice care.

### Supplementary information

**Additional file 1: APPENDIX 3.** Critical Appraisal Tool

## Data Availability

All data generated or analysed during this study are included in this published article.

## **APPENDIX 1:** PRISMA Checklist

**Table:**

Section/topic	#	Checklist item	Reported on page #
**TITLE**			
Title	1	Identify the report as a systematic review, meta-analysis, or both.	1
**ABSTRACT**			
Structured summary	2	Provide a structured summary including, as applicable: background; objectives; data sources; study eligibility criteria, participants, and interventions; study appraisal and synthesis methods; results; limitations; conclusions and implications of key findings; systematic review registration number.	2, 3
**INTRODUCTION**			
Rationale	3	Describe the rationale for the review in the context of what is already known.	4–6
Objectives	4	Provide an explicit statement of questions being addressed with reference to participants, interventions, comparisons, outcomes, and study design (PICOS).	6
**METHODS**			
Protocol and registration	5	Indicate if a review protocol exists, if and where it can be accessed (e.g., Web address), and, if available, provide registration information including registration number.	
Eligibility criteria	6	Specify study characteristics (e.g., PICOS, length of follow-up) and report characteristics (e.g., years considered, language, publication status) used as criteria for eligibility, giving rationale.	6, 7
Information sources	7	Describe all information sources (e.g., databases with dates of coverage, contact with study authors to identify additional studies) in the search and date last searched.	6
Search	8	Present full electronic search strategy for at least one database, including any limits used, such that it could be repeated.	29
Study selection	9	State the process for selecting studies (i.e., screening, eligibility, included in systematic review, and, if applicable, included in the meta-analysis).	7
Data collection process	10	Describe method of data extraction from reports (e.g., piloted forms, independently, in duplicate) and any processes for obtaining and confirming data from investigators.	8
Data items	11	List and define all variables for which data were sought (e.g., PICOS, funding sources) and any assumptions and simplifications made.	8
Risk of bias in individual studies	12	Describe methods used for assessing risk of bias of individual studies (including specification of whether this was done at the study or outcome level), and how this information is to be used in any data synthesis.	7
Summary measures	13	State the principal summary measures (e.g., risk ratio, difference in means).	8
Synthesis of results	14	Describe the methods of handling data and combining results of studies, if done, including measures of consistency (e.g., I^2^) for each meta-analysis.	8

Page 1 of 2.

**Table:**

Section/topic	#	Checklist item	Reported on page #
Risk of bias across studies	15	Specify any assessment of risk of bias that may affect the cumulative evidence (e.g., publication bias, selective reporting within studies).	
Additional analyses	16	Describe methods of additional analyses (e.g., sensitivity or subgroup analyses, meta-regression), if done, indicating which were pre-specified.	
**RESULTS**			
Study selection	17	Give numbers of studies screened, assessed for eligibility, and included in the review, with reasons for exclusions at each stage, ideally with a flow diagram.	8
Study characteristics	18	For each study, present characteristics for which data were extracted (e.g., study size, PICOS, follow-up period) and provide the citations.	8, 9, 16, 17
Risk of bias within studies	19	Present data on risk of bias of each study and, if available, any outcome level assessment (see item 12).	17–20
Results of individual studies	20	For all outcomes considered (benefits or harms), present, for each study: (a) simple summary data for each intervention group (b) effect estimates and confidence intervals, ideally with a forest plot.	17–20
Synthesis of results	21	Present results of each meta-analysis done, including confidence intervals and measures of consistency.	17–20 (narrative synthesis)
Risk of bias across studies	22	Present results of any assessment of risk of bias across studies (see Item 15).	
Additional analysis	23	Give results of additional analyses, if done (e.g., sensitivity or subgroup analyses, meta-regression [see Item 16]).	
**DISCUSSION**			
Summary of evidence	24	Summarize the main findings including the strength of evidence for each main outcome; consider their relevance to key groups (e.g., healthcare providers, users, and policy makers).	11–13
Limitations	25	Discuss limitations at study and outcome level (e.g., risk of bias), and at review-level (e.g., incomplete retrieval of identified research, reporting bias).	13, 14
Conclusions	26	Provide a general interpretation of the results in the context of other evidence, and implications for future research.	14, 15
**FUNDING**			
Funding	27	Describe sources of funding for the systematic review and other support (e.g., supply of data); role of funders for the systematic review.	22

*From:* Moher D, Liberati A, Tetzlaff J, Altman DG, The PRISMA Group (2009). Preferred Reporting Items for Systematic Reviews and Meta-Analyses: The PRISMA Statement. PLoS Med 6(7): e1000097. doi:10.1371/journal.pmed1000097

For more information, visit: **www.prisma-statement.org**.

Page 2 of 2

## APPENDIX 2

Search strategy: PubMed

1. “Patients” [Mesh]
2. “Research Subjects” [Mesh]
3. “Men” [Mesh]
4. “Women” [Mesh]
5. “Visitors to Patients” [Mesh]
6. 1 OR 2 OR 3 OR 4 OR 5
7. “Stress, Psychological” [Mesh]
8. “Stress, Physiological” [Mesh]
9. “Anxiety” [Mesh]
10. Distress
11. Strain
12. 7 OR 8 OR 9 OR 10 OR 11
13. 6 AND 12
14. “patient stress”
15. 13 OR 14
16. “Compassion Fatigue” [Mesh]
17. “Burnout, Professional” [Mesh]
18. “Mental Disorders” [Mesh]
19. 16 OR 17 OR 18
20. 15 NOT 19
21. “Environment” [Mesh]
22. “Health Facilities” [Mesh]
23. 21 OR 22
24. 20 AND 23

Search strategy: PsycInfo

1. “Anxiety” [Index Term]
2. “Chronic Stress” [Index Term]
3. “Distress” [Index Term]
4. “Environmental Stress” [Index Term]
5. “Financial Strain” [Index Term]
6. “Physiological Stress” [Index Term]
7. “Psychological Stress” [Index Term]
8. “Stress” [Index Term]
9. “Stress Reactions” [Index Term]
10. 1 OR 2 OR 3 OR 4 OR 5 OR 6 OR 7 OR 8 OR 9
11. “Environment” [Index Term]
12. “Environmental Enrichment” [Index Term]
13. “Facility Environment” [Index Term]
14. “Learning Environment” [Index Term]
15. “Therapeutic Environment” [Index Term]
16. 11 OR 12 OR 13 OR 14 OR 15
17. 10 AND 16
18. Any Field: “Journal”
19. Year: 1997 to 2007
20. Population Group: Human
21. 17 AND 18 AND 19 AND 20

Search strategy: CINAHL

1. MH “Patients+”
2. MH “Stress+”
3. 1 AND 2
4. “patient stress”
5. 3 OR 4
6. MH “Caregiver Burden”
7. MH “Compassion Fatigue”
8. MH “Stress, Occupational+”
9. 6 OR 7 OR 8
10. 5 NOT 9
11. MH “Health Facilities+”
12. 10 AND 11

Search strategy: Embase

1. ‘stress’/exp.
2. ‘patient’/exp.
3. 1 AND 2
4. ‘patient stress’
5. 3 OR 4
6. ‘burnout’/exp.
7. ‘caregiver burden’/exp.
8. ‘job stress’/exp.
9. 6 OR 7 OR 8
10. 5 NOT 9
11. ‘health care facility’/exp
12. 10 AND 11
13. [1997–2017]/py
14. [humans]/lim
15. [English]/lim
16. [article]/lim
17. 12 AND 13 AND 14 AND 15 AND 16

## Abbreviations

CCU: Critical care unit
DV: Dependent variable
GCU: General care unit
HCP: Health care professional
ICU: Intensive care unit
NICU: Neonatal intensive care unit
PHDU: Paediatric high dependency unit
PICU: Paediatric intensive care unit
PSS: Parental stressor scale
SD: Standard deviation
