# Supplementary material for: The impact of health literacy environment on patient stress: a systematic review
Source: BMC Public Health. 2020 May 24;20:749. doi: 10.1186/s12889-020-08649-x (PMC7245697; doi:10.1186/s12889-020-08649-x)
Supplement: Supplementary file 1 — Additional file 1: APPENDIX 3. Critical Appraisal Tool [file 12889_2020_8649_MOESM1_ESM.pdf]

APPENDIX 3: Critical Appraisal Tool

THE IMPACT OF HEALTH LITERACY ENVIRONMENT ON PATIENT STRESS<br><br>CRITICAL APPRAISAL OF EVIDENCE OF EFFECTIVENESS

Reviewer: ______________________________ **Date**: ________________

Author: ______________________________ **Year**: ____________ **Record Number**: ___________

SCREENING QUESTIONS

I. Is the study one of the following quantitative designs?

Experimental/Quasi-Experimental. Includes treatment/intervention

RCT ☐ Controlled Trial ☐ Controlled Before and After ☐ Interrupted Time Series ☐ Pre-post study ☐

Non-experimental. No treatment/intervention

Cohort study ☐ Case Control ☐ Case study or case series ☐ Cross-sectional survey ☐

II. Is the study one of the following qualitative designs?

Generalisable studies ☐ Conceptual studies ☐ Descriptive studies ☐ Single case studies ☐

III. If the study does not include data collection and analysis: **STOP NOW** ☐

If the study is an empirical study, appraise the quality of the reported research using the questions below.

APPRAISAL QUESTIONS

Q No.	Appraisal Question	Yes	No	Can't tell or Mixed response
1	Is the purpose of the study clear and well defined?	2	0	1
2	Is the population well defined and properly selected?	2	0	1
3	Are the methods clearly described and appropriate for the type of study reported?	2	0	1
4	Are the results presented in a clear and understandable format?	2	0	1
5	Does the interpretation of the results seem consistent with the results presented?	2	0	1
6	Are there any other explanations that could account for these results?	0	2	1
	Total score:			*(Maximum of 12)*

**RECOMMENDATION:**	Include ☐	Exclude ☐	Seek further information ☐

Comments (including reasons for exclusions): ____

(Questions derived from Oxman & Guyatt, 1994; JBI Criteria;
